# Advanced technologies for biomedical applications by emerging researchers in Asia‐Pacific

**DOI:** 10.1002/btm2.10621

**Published:** 2023-11-19

**Authors:** Weiping Wang, Chenjie Xu, Jin‐Wook Yoo

**Affiliations:** ^1^ Department of Pharmacology and Pharmacy, Li Ka Shing Faculty of Medicine The University of Hong Kong Hong Kong SAR China; ^2^ Department of Biomedical Engineering City University of Hong Kong Hong Kong SAR China; ^3^ College of Pharmacy and Research Institute for Drug Development Pusan National University Busan South Korea

**Translational Impact Statement**

Researchers from the Asia‐Pacific region are making significant contributions to the progress of biomedical engineering. In this special issue, we have collected innovative research articles from emerging investigators in the Asia‐Pacific region. These articles showcase the latest advancements in technology for biomedical applications, focusing on drug delivery, disease diagnosis, and the invention of biomedical devices. The article is aimed to offer valuable insights and keep readers well‐informed in the ever‐changing field of biomedical engineering.

## 1 INTRODUCTION

Over the past few decades, there have been numerous breakthroughs in advanced technologies for biomedical applications. These advancements have formed the foundation for emerging fields such as RNA therapeutics, gene editing, and immunotherapy, enabling the resolution of various medical challenges, including COVID‐19, genetic diseases, malignant tumors, and more. Furthermore, state‐of‐the‐art biomedical technologies, including intelligent drug delivery systems, diagnostic imaging agents, and smart medical devices, are continually developed to prevent and treat life‐threatening diseases from the bench to the bedside.

Researchers in the Asia‐Pacific region are making substantial contributions to this burgeoning field. A number of advanced biological therapeutics are first approved in Asia‐Pacific countries, with numerous innovative pipelines undergoing preclinical or clinical‐stage development. This special issue of *Bioengineering & Translational Medicine* (Volume 8, Issue 6) aims to showcase the innovative research originating from Asia‐Pacific investigators in the fields of drug delivery, diagnosis, and medical devices. The presented advanced technologies for biomedical applications possess the potential to impact clinical practice.

## 2 DRUG DELIVERY

Drug delivery technologies help convert potential therapeutics into commercially valuable pharmaceutical products.^1^ Today there are a wide range of therapeutics including small molecules, proteins and peptides, antibodies, nucleic acids, as well as live cells. For example, Wang et al. decorated the photosensitizer tetraphenylethylene (TPE) with a cationic group and oligomeric ethylene glycol group, and the prepared compound OEO‐TPE‐MEM possesses superior antibacterial effects and biosafety than TPE.^2^ Tamura et al. developed a new therapeutic method utilizing CRISPR/Cas9‐edited human‐induced pluripotent stem cells to treat malignant glioma.^3^ However, some of these therapeutics are instable or quickly excreted in vivo while some cannot reach the target by themselves.

The development of drug delivery systems is to tackle the mentioned challenges. Various drug delivery systems have been developed to precisely deliver small molecules to the right place while minimizing their off‐target retention. For example, Zhang et al. synthesized halofuginone‐loaded mesoporous platinum (mPt) nanoparticles for treating breast cancer. They successfully enhanced the therapeutic efficacy by combining the photothermal therapy of mPt nanocarriers and the extracellular matrix‐remodeling effects of halofuginone.^4^ Yoo et al. prepared the thiamine pyrophosphate‐decorated human serum albumin nanoclusters adsorbing doxorubicin or methotrexate to achieve the synergistic anti‐tumor effects with cisplatin for osteosarcoma therapy.^5^ The electric‐pulse‐driven nanopore‐electroporation system using the needle electrode was developed by Lee et al., which enables the precise and targeted intracellular delivery of small molecules into deep tissues.^6^ Luo et al. incorporated tannic acid, a naturally derived antimicrobial agent, into electrospun polyvinyl alcohol fibers for the healing of infected wounds.^7^ Yang et al. fabricated the peroxide‐derived AgAu‐based nanoboxes to treat *Clostridioides difficile* infection.^8^ Le et al. also reported an enzyme‐responsive macrocyclam‐metal complex to deliver gadolinium to tumor tissues for magnetic resonance imaging.^9^

Besides targeted delivery, drug delivery systems can also improve the in vivo stability of peptides, protein, nucleic acids, and even live cells. For example, Wu et al. modified strain *Escherichia coli* Nissle 1917 by CRISPR/Cas9‐mediated genome editing to obtain a novel strain, which showed neuroprotective effects against Parkinson's disease by delivering glucagon‐like peptide‐1 and restoring the disturbance of gut microbiota.^10^ Chang et al. employed cryomicroneedles to store and transdermally deliver dendritic cells and anti‐PD‐1 antibody, leading to the successful therapy of melanoma in a mouse model.^11^ Liao et al. applied ultrasound with microbubbles to improve the delivery of insulin‐like growth factor 1 into inner ears.^12^ Jeon et al. fabricated a fucoidan‐based complex coacervate‐laden injectable hydrogel that enabled efficient entrapment and gradual release of interleukin‐2, resulting in the enhancement of T‐cell‐based immunotherapies.^13^ Kim et al. designed a hybrid nanorod/nanodisk gel network that encapsulated indocyanine green (ICG), glucose oxidase (GOx), and copper (II) sulfate for synergetic tumor chemodynamic/starvation/photothermal therapy.^14^ Banstola et al. prepared ROS‐responsive thioketal polymer‐based nanoparticles for the simultaneous delivery of small molecules (doxorubicin and R848) and macrophage inflammatory protein‐3 alpha, which were effective in treating breast cancer by inducing immunogenic cell death.^15^ Zhang et al. genetically fabricated the nano‐melittin vesicles, which can further encapsulate chemotherapeutic drugs or nanoparticles for synergetic cancer therapy.^16^ Zhu et al. designed various nanobodies of anti‐vascular endothelial growth factor (VEGF) proteins for sustained release of proteins, which provides a novel approach for chronic intravitreal administration of anti‐VEGF proteins.^17^ Phung and colleagues prepared polymer‐lipid hybrid nanoparticles that co‐encapsulated LTX‐315, a pioneering oncolytic cationic peptide, and TGF‐β1 siRNA to enhance the effectiveness of cancer immunotherapy.^18^ Cai et al. prepared gold nanoparticles conjugated with internalizing‐RGD peptide and loaded with siCDK7 for inducing immunotherapeutic responses against lung cancer.^19^ Kim et al. prepared piperazine‐based ionizable lipid nanoparticles for the targeted delivery of mRNA to fibrotic lungs. This innovative approach enhanced the potency and safety of mRNA delivery compared with conventional lipid nanoparticles.^20^ Xu et al. developed a living prosthetic breast in the form of injectable gelatin methacryloyl microspheres. Zeolitic imidazolate framework (ZIF) nanoparticles loaded with urolithin C and adipose‐derived stem cells are encapsulated in the microspheres to inhibit tumor recurrence and promote tissue regeneration simultaneously.^21^ For the delivery of live cells, the nanostructured hydroxyapatite/alginate composite hydrogel was developed for the intestinal delivery of live microorganisms, and the hydrogel‐based microdevice was prepared for the co‐encapsulation of therapeutic microtissues and pro‐angiogenic endothelial cells.^22^, ^23^

## 3 DISEASE DIAGNOSIS

Disease diagnosis involves the detection and quantification of specific biological molecules that are closely related with the presence of a pathogen or the disease progress. Lin et al developed a phase‐sensitive surface plasmon resonance biosensor for the fast and accurate detection of severe acute respiratory syndrome coronavirus 2.^24^ Zheng et al designed a skin patch composing of swellable microneedles and electrochemical test strips to monitor the levels of glucose and alcohol inside the body.^25^ Li et al. reported a wearable wound dressing system for monitoring wound condition and sepsis‐related biomarker procalcitonin in a real‐time manner, which showed great potential for early sepsis diagnosis.^26^

A significant trend is the combination of medical diagnosis techniques with the power of artificial intelligence (AI), machine learning and deep learning. For example, Tang et al. utilized an AI‐empowered rapid object detector for real‐time feedback control of magnetic digital microfluidics, which achieved automated in vitro diagnostics.^27^ Tostado et al. developed an AI‐assisted single‐cell phenomic‐transcriptomic platform, which showed great potential in elucidating mechanisms and therapeutic targets against immune response.^28^ Misra et al. introduced novel deep learning‐based techniques for segmenting lesions and distinguishing between benign and malignant cases by analyzing B‐mode and strain elastography images.^29^ In another research direction, Yuan et al. utilized machine learning methods to predict the permeation of drugs through the microneedle‐treated skin.^30^

## 4 BIOMEDICAL DEVICES

Besides drug delivery and disease diagnosis, there are also many advancements in the development of medical devices, leading to significant improvements in patient outcomes and overall quality of life. For example, Tan et al. designed a palmar‐side hand protector, which protected hands from microbial contamination like traditional gloves but significantly improved comfort and inconspicuousness.^31^ Song et al. and Koo et al. developed novel biomedical devices for the collection of saliva and the isolation of urinary circulating RNAs, respectively, resulting in a significant improvement in analytical performance.^32^, ^33^ Yu et al. prepared innovative acellular nerve allografts (ANAs), which could retain a higher concentration of extracellular matrix bioactive molecules and regenerative factors while efficiently eliminating cellular antigens. Applying these ANAs in peripheral nerve regeneration holds promise for clinical use.^34^ Yu et al. engineered a structurally organized bladder construct by integrating buccal mucosa graft with vascularized smooth muscle tissue, which facilitated the repair of functional bladder defects.^35^

## 5 CONCLUSIONS

This special issue emphasizes the cutting‐edge technological advancements from the Asia‐Pacific region for biomedical applications like drug delivery and disease diagnosis. We hope that this collection provides readers with valuable insights and inspires them to pursue further research to develop innovative technologies for biomedical applications.
